# The alerting effect of the wake maintenance zone during 40 hours of sleep deprivation

**DOI:** 10.1038/s41598-018-29380-z

**Published:** 2018-07-20

**Authors:** Jan de Zeeuw, Sophia Wisniewski, Alexandra Papakonstantinou, Frederik Bes, Amely Wahnschaffe, Mandy Zaleska, Dieter Kunz, Mirjam Münch

**Affiliations:** 1 Charité – Universitätsmedizin Berlin, corporate member of Freie Universität Berlin, Humboldt-Universität zu Berlin, and Berlin Institute of Health, Institute of Physiology, Sleep Research & Clinical Chronobiology, Berlin, Germany; 2 Charité – Universitätsmedizin Berlin, corporate member of Freie Universität Berlin, Humboldt-Universität zu Berlin, and Berlin Institute of Health, Institute of Medical Immunology, Laboratory of Chronobiology, Berlin, Germany; 3 Clinic for Sleep & Chronomedicine, St. Hedwig-Krankenhaus, Berlin, Germany; 4 Intellux GmbH, Berlin, Germany

## Abstract

Under entrained conditions, the accumulation of homeostatic sleep pressure in the evening is opposed by a strong circadian arousal signal prior to the dim light melatonin onset, called the Wake Maintenance Zone (WMZ). This study aimed at investigating the impact of the WMZ on different cognitive performance tests, as well as on subjective and objective sleepiness. Twelve young male participants completed a constant routine protocol with 40 h of extended wakefulness that included two WMZs. Cognitive tests and saliva samples were assessed hourly, while the electroencephalogram (EEG) was recorded continuously. Participants improved in cognitive response inhibition during WMZ1 (13.5 h awake) and sustained attention during WMZ2 (37.5 h awake), but not in higher executive function tests. There were significant EEG power density reductions in the delta/theta frequency range during WMZ1 and in delta/theta, alpha, and sigma/beta ranges during WMZ2, with a greater change in the sigma/beta range during WMZ2 compared to WMZ1. EEG power reductions coincided during WMZ1 with stable subjective sleepiness and sustained attention. During WMZ2, EEG power reductions were more pronounced and coincided with improved sustained attention. Our results suggest the circadian arousal signal in the evening differently modulates cognitive functions and EEG power depending on the duration of prior wakefulness.

## Introduction

Mammalian sleep-wake regulation undergoes modulation by two main processes. A homeostatic process (Process S) accounts for the build-up of sleep pressure, i.e. it increases with time awake. This can be assessed by the increase of slow frequency EEG activity during wakefulness and its decline during the following sleep period^1–4^. However, sleep pressure does not linearly increase over time^5^, even after extended wakefulness by partial or total sleep deprivation (as reviewed by Schmidt *et al*.^6^). Instead, sleep propensity (the tendency to fall asleep) is also modulated by a circadian process. The circadian component (Process C) regulates the endogenous circadian rhythm of sleep-wake regulation across 24 hours^1^ and is governed by the biological clock in the suprachiasmatic nucleus of the hypothalamus (SCN)^7^. Both processes interact with each other and can only be determined separately by means of specialized protocols such as the forced desynchrony protocol^8^ or nap paradigms^9^. When analyzing the circadian impact on sleep propensity under these conditions, the circadian drive for sleep is very low in the early evening before bedtime, shortly before the secretion onset of the pineal hormone melatonin^8^. At first glance, this may seem counterintuitive, because it implies a high circadian drive for alertness in the evening^8–12^. Yet, it was shown that the circadian arousal signal in the early evening leads to higher subjective and objective alertness and is thus opposing the accumulated homeostatic sleep pressure - a phenomenon called the ‘wake maintenance zone’ (WMZ) or ‘forbidden zone of sleep’^5,9,11^. The dynamic interaction of both processes in sleep-wake regulation enables a consolidated wake period of approximately 16 hours during daytime and a consolidated sleep period at night in humans^13,14^.

In addition to sleep-wake regulation, most physiological and behavioral processes undergo homeostatic and circadian regulation. Cognitive performance, as well as subjective and objective alertness, is also high during the WMZ in the evening^15–24^. Significant performance improvements of sustained attention occur during the WMZ, both after a normal duration of prior wakefulness as well as after sleep deprivation^16^. Better working memory performance during the WMZ has been linked to sleep-dependent higher hypothalamic activation as assessed in the Blood Oxygenation Level Dependent (BOLD) response of the MRI after a normal duration of prior wakefulness, but not after very low or very high sleep pressure conditions^25^. These studies suggest a differential impact on cognitive performance during the WMZ, depending on the duration of prior wakefulness.

We aimed at measuring different domains of cognitive performance, such as sustained attention, executive functions and working memory, as well as subjective and objective sleepiness during 40 hours of extended wakefulness and under controlled conditions (i.e. constant routine^26^; CR). The experiment was designed to include two WMZs. We hypothesized that not all cognitive functions are similarly altered over the course of the protocol and specifically not during the two WMZs. In addition, we expected differences in subjective and objective sleepiness between both WMZs, which depend on the prior duration of wakefulness.

## Results

### Cognitive performance

#### Time course of cognitive performance during 40 hours of extended wakefulness

**Figure 1:**
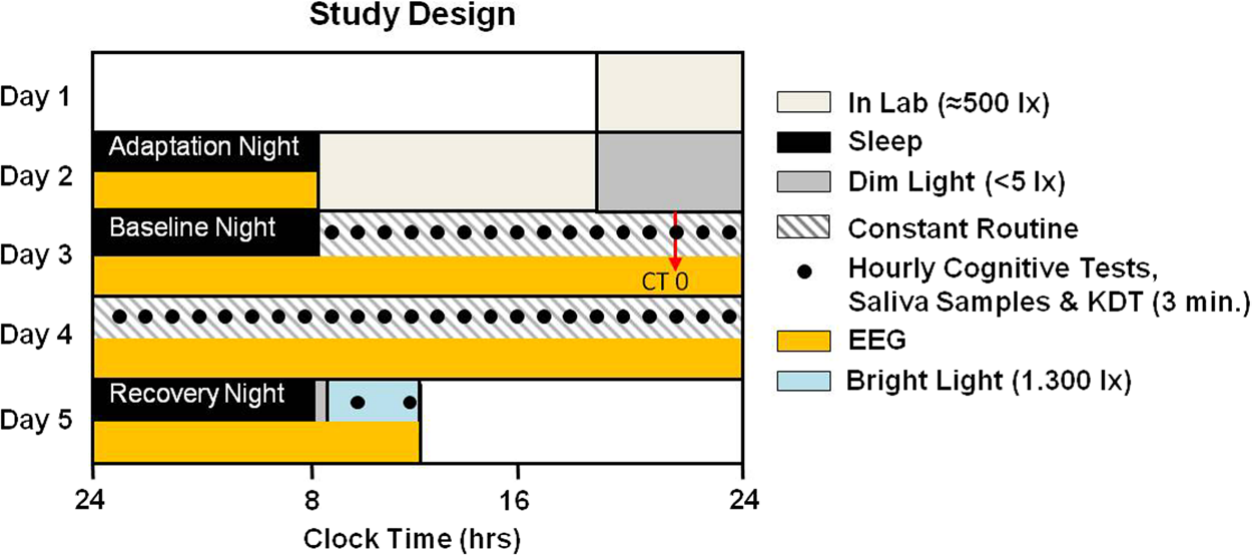
Study Design. Participants arrived at the laboratory 3 h prior to habitual bedtime on day 1 and slept in the laboratory at their habitual bed- and wake-times (=adaptation night). On day 2 participants stayed in the laboratory where they were free to move in their room. Following the baseline night, the 40 h CR protocol with constant wakefulness in bed started at habitual wake time on day 3. During the CR, cognitive tests and Karolinska Drowsiness Tests (KDTs; 3 min; open eyes) were performed hourly. Salivary samples for hormonal analyses were also collected hourly. The first cognitive tests were performed at CT -13, which was 13 h prior to the DLMO of the first evening of the CR (DLMO = CT 0; red arrow). The CR protocol ended at CT 26. After the recovery night on day 5 participants were exposed to 3 h of bright light (for purposes not reported here) and were free to leave the laboratory about 4 h after habitual wake time.

**Figure 2:**
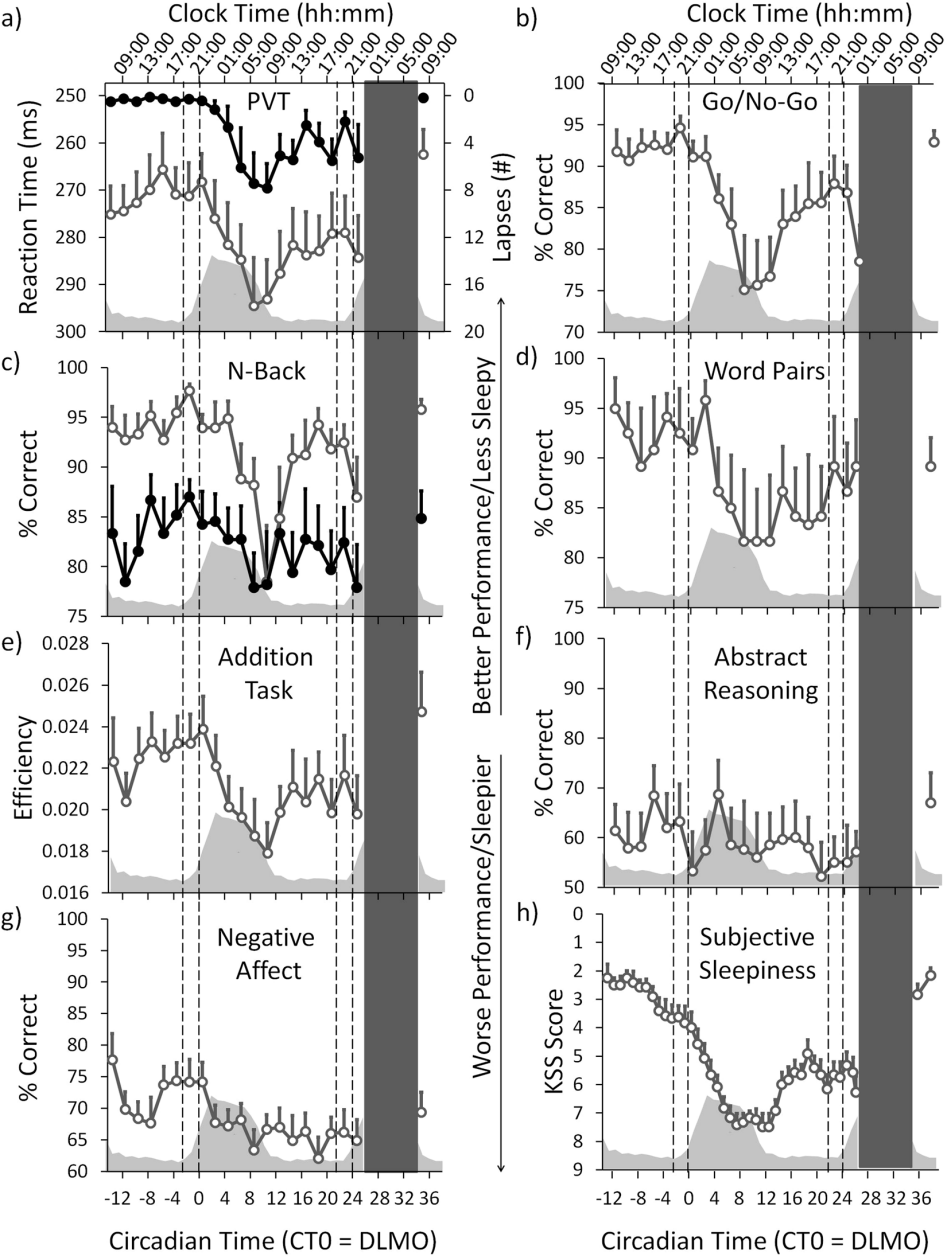
Cognitive performance and subjective sleepiness. The upper x-axis shows the mean clock time. The lower x-axis shows the circadian time in hours relative to the DLMO of the first evening (= CT 0). Note: the y-axis for PVT performance and the KSS have been inverted so that the direction is similar to the other cognitive tests (i.e. higher indicates better performance and less sleepy). The grey inlay shows the melatonin secretion profile across the CR and during the first 4 h after the recovery night. The two sets of dotted lines indicate the time ranges of both WMZs. The black bar represents the scheduled sleep episode (=recovery night). Mean ± SEM (n = 12). See also Table S1.1. (**a**) PVT; open grey circles = median reaction times; filled circles = lapses. (**b**) Go/No-Go Test. (**c**) N-back Test; open grey circles = 2-Back version; filled circles = 3-Back version (ns). (**d**) Word-Pair Memory Test (ns). (**e**) Addition Task. (**f**) Abstract Reasoning (ns). (**g**) Negative Affect Test. (**h**) Subjective Sleepiness (KSS).

The time course of all results was expressed as circadian time (CT) relative to the individual timing of the dim light melatonin onset (DLMO) in the first evening of the CR (= CT 0; see also Fig. 1 and Methods) which occurred on average at 21:17 (±1:09 h; SD). For the time course across 40 h of extended wakefulness there was a significant modulation in all but three cognitive tests (Psychomotor Vigilance Test (PVT) median reaction times: F_1,20_ = 4.042, p < 0.001; PVT lapses: F_1,20_ = 3.208, p < 0.001; Go/No-Go: F_1,20_ = 4.815, p < 0.001; 2-back: F_1,20_ = 3.519, p < 0.001; Addition Task: F_1,20_ = 3.611, p < 0.001; Negative Affect: F_1,20_ = 2.810, p < 0.001; main effects of TIME; Fig. 2a–c,e,g; Table S1.1). Post-hoc comparisons between time points for each test (corrected for multiple comparisons, see Methods) revealed a very similar time course for the PVT, Go/No-Go and the Addition Task with best performance during the first biological day (i.e. CT −13 until CT 0; Fig. 2a–c and e). Immediately following the DLMO (i.e. CT 0), performance on the PVT, Go/No-Go, and Addition Task gradually declined, leading to significantly lower levels during the late biological night/early morning (CT 7 until CT 13), when compared to the first biological day. Compared to the late night/early morning, performance significantly improved during the second day (starting at CT 15). Results for the 2-Back test showed a similar time course, except that cognitive performance on the first day remained at high levels for longer (until CT 5; Fig. 2c). The more difficult version of the N-Back test (3-Back) showed no significant change over time; neither did performance in the Word-Memory Test or the Abstract Reasoning Test (3-Back: F_1,20_ = 1.124, p = 0.336; Word-Memory: F_1,20_ = 1.109, p = 0.349; Abstract Reasoning: F_1,20_ = 0.789, p = 0.722; Fig. 2c,d and f).

The time course for correctly recognized negative emotions (=Negative Affect Test) showed a significant modulation that differed from the other tests (F_1,20_ = 2.810, p < 0.001; Fig. 2g). Here, performance was high at the very start of the CR (at CT −13) and declined during the morning and early afternoon. In the late afternoon (CT −5), performance improved and remained high until the evening (CT 1). It then decreased again and, compared to the time between CT −5 and CT 1, performance remained significantly lower for the rest of the CR.

#### Cognitive performance changes during the two wake maintenance zones

The two WMZs were defined as the 3-hour interval prior to DLMO. We analyzed the changes during the WMZs in three ways (see also Fig. S1): First, we compared absolute performance levels between the hours directly prior to the WMZs (PH 1 vs. PH 2) and we compared the absolute performance levels during the two WMZs with each other (WMZ 1 vs. WMZ 2). Secondly, we compared the performance during each WMZ with the hour preceding it (WMZ 1 vs. PH 1; and WMZ 2 vs. PH 2). Lastly, we determined whether extended wakefulness had a differential impact on performance changes during the WMZs by comparing the changes in performance between the two WMZs (i.e. while each WMZ was expressed relative to its prior hour; WMZ 1/PH 1 vs. WMZ 2/PH 2).

Comparison of the absolute performance levels between the two WMZs showed significantly more PVT lapses (p = 0.047, Cohen’s d effect size = 0.968) as well as less accuracy on the Go/No-Go (F_1,11_ = 7.084, p = 0.022, d = −0.747) and the 2-Back Test (F_1,16_ = 13.921, p = 0.002, d = −1.160) during the second WMZ (=WMZ 2; after 37.5 h awake) compared to the first WMZ (=WMZ 1; after 13.5 h awake; Fig. 2a–c). The other performance tests did not show significant differences between both WMZs. In the hour prior to WMZ 2, performance was significantly worse compared to the hour prior to WMZ 1 for PVT lapses, the Go/No-Go, the 3-Back Test, the Word-Memory Test and the Addition Task (respectively: p = 0.008, d = 1.108; F_1,10_ = 6.774, p = 0.027, d = −0.697; F_1,13_ = 8.180, p = 0.014, d = −0.804; F_1,11_ = 10.729, p = 0.007, d = −1.135; Fig. 2a–e; Table S1.2.1).

Comparing the performance during each WMZ with the hour immediately prior to the respective WMZ, we found that during WMZ 1 (i.e. at CT −2) the Go/No-Go was the only cognitive test which showed a significant performance improvement compared to the test taken prior to WMZ 1 (i.e. CT −4; F_1,12_ = 5.142, p = 0.043, d = 0.380; Fig. 2b). During WMZ 2 (i.e. CT 23) there were significantly less PVT lapses compared to the test taken prior to WMZ 2 (i.e. CT 21; p = 0.012, d = −0.687; Fig. 2a; Table S1.2.2).

Then, we calculated and compared the cognitive performance changes of the two WMZs (with each WMZ was expressed relative to its prior hour). This showed a significant greater decrease in PVT lapses (p = 0.009, d = −0.986) and a greater increase of efficiency in the Addition Task (F_1,11_ = 6.843, p = 0.025, d = 0.808) during WMZ 2, when compared to WMZ 1 (Table S1.2.3). The changes in other performance tests did not show significant differences between both WMZs.

#### Cognitive performance after the recovery night

After the 8 h recovery night and during the polychromatic bright light exposure (see Methods), cognitive performance improved again to levels similar to those at the beginning of the CR protocol for all tests, except for PVT median reaction times: these were significantly faster than during the first 6 h of the CR (p < 0.05; Fig. 2a–g).

### Subjective and objective sleepiness

#### Time course of subjective and objective sleepiness across 40 hours of extended wakefulness

Subjective sleepiness showed a significant change over time (main effect of TIME; F_1,41_ = 8.596, p < 0.001; Fig. 2h; Table S2.1). Post-hoc tests showed that participants rated themselves least sleepy during the first morning and early afternoon of the CR (CT −13 until CT −7). Compared to the morning and early afternoon, participants were significantly sleepier in the late afternoon (starting at CT −5) and became most sleepy in the late night/early morning hours (CT 7 until CT 12). During the second day, participants were significantly less sleepy (starting at CT 15) compared to the late night/early morning. However, compared to the first day (CT −13 until CT 0), participants remained significantly sleepier during the entire second day (i.e. until CT 26; p < 0.05).

**Figure 3:**
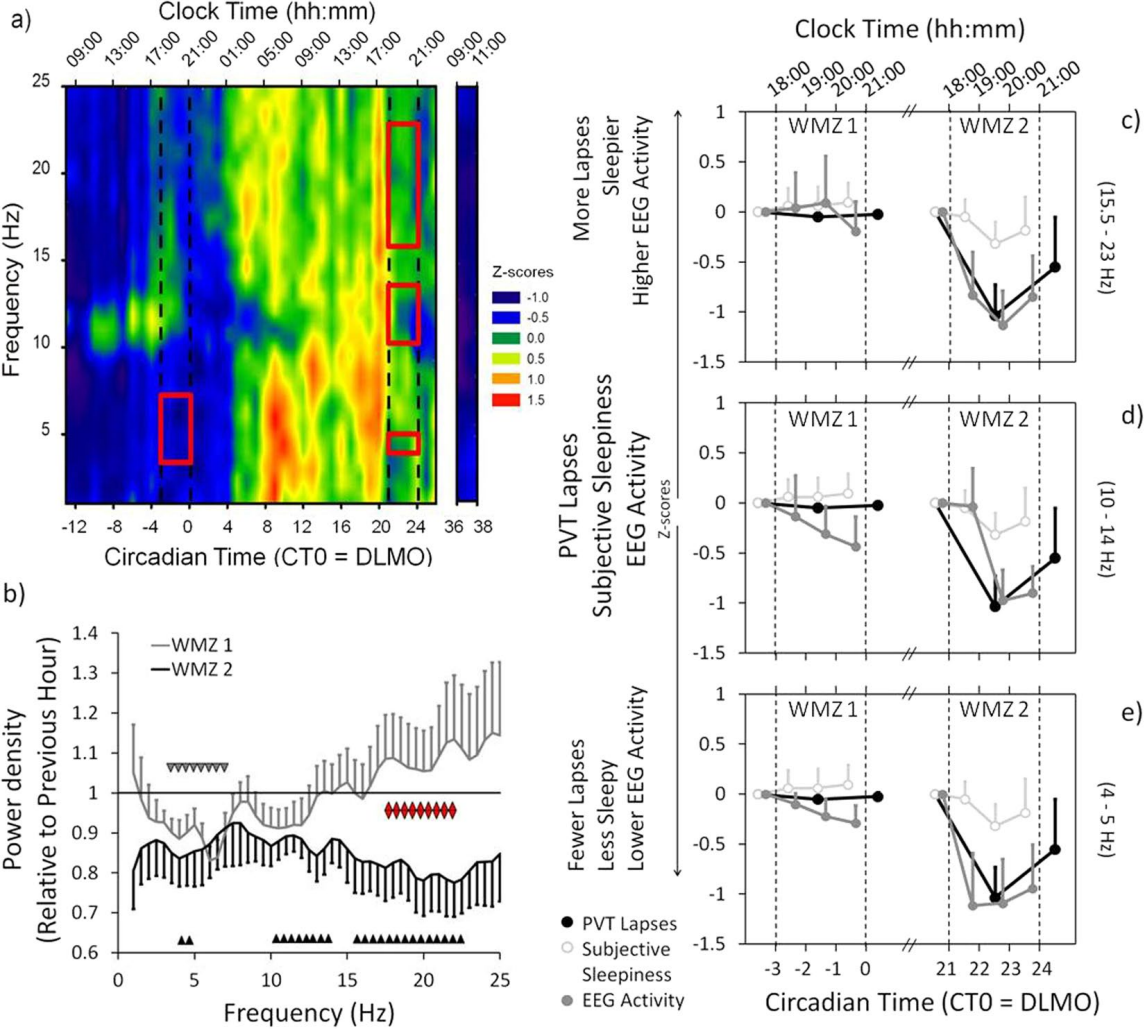
Wake-EEG, PVT lapses and subjective sleepiness during the WMZs. (**a**) Heat plot of the wake-EEG power density (standardized data; 0.5 to 25 Hz) over the 40 h CR and during the 2 h after the recovery night (frontal derivation; F4). Blue colors = lower EEG power density. Red colors = higher EEG power density. The upper x-axis shows the mean clock time (hh:mm). The lower x-axis shows the circadian time in hours relative to the DLMO of the first evening (=CT 0). The two sets of dotted lines indicate the time ranges of the two WMZs. The red rectangles show the frequency bins with significantly lower EEG power density during the WMZ compared to the preceding hour (WMZ 1: delta/theta range 3.0–7.0 Hz; WMZ 2: delta/theta 4.0–5.0 Hz, alpha 10.0–14.0 Hz and sigma/beta 15.5–23.0 Hz). (**b**) Changes in EEG power density during WMZ 1 (grey line) and WMZ 2 (black line) relative to the preceding hour. Frequency bins with significantly lower power density during WMZ 1 are indicated by grey downward triangles and during WMZ 2 by black upward triangles. Red triangles show the frequency bins (high sigma/beta range 17.5–22.5 Hz) with statistically significant differences between both WMZs (while expressed relative to the preceding hour). (**c**, **d** & **e**) All three panels show subjective sleepiness (open circles) and PVT lapses (closed black circles) during both WMZs relative to the preceding hour. Results for WMZ 1 are shown on the left and for WMZ 2 on the right side. Each panel compares the change in subjective sleepiness and PVT performance with one of the EEG frequency ranges (closed grey circles) which showed a reduction in EEG power density during WMZ 2 (from top to bottom: sigma/beta, alpha and delta/theta ranges). The upper x-axis shows the mean clock time. The lower x-axis shows the circadian time in hours relative to DLMO. The two sets of dotted lines indicate the time ranges of the WMZs. All data was standardized (z-scores). Mean ± SEM.

For objective sleepiness, the waking EEG power density of the 3 min Karolinska Drowsiness Test (KDT; see Methods) showed a significant change over time for all 0.5 Hz bins in the range of 0.5–25 Hz (p < 0.001; Fig. 3a). Here, we report the results of the frontal derivation (F4), but similar results were found in central and occipital derivations (data not shown). Special interest was given to those EEG frequency ranges which showed a significant change in EEG power density during the WMZs (EEG delta/theta 4–5 Hz: F_41,232_ = 8.596, p < 0.001; alpha 10–14 Hz: F_41,208_ = 2.861, p < 0.001; and sigma/beta activity 15.5–23 Hz: F_41,213_ = 2.607, p < 0.001; Table S2.1; see also below). Post-hoc testing indicated lowest EEG delta/theta activity (4–5 Hz) during the first morning (CT −13 until CT −7; Fig. 3a). It then increased significantly during night time (starting at CT 5) and remained higher during the entire second day (i.e. all time points after CT 5) compared to the first morning. EEG delta/theta activity showed two statistically determined maxima, one in the morning (at CT 10) and one in the late afternoon (at CT 20; p < 0.05).

EEG alpha (10–14 Hz) and sigma/beta activity (15.5–23 Hz) were also at their lowest level at the beginning of the CR (CT −13 until CT −11; Fig. 3a). Compared to these low levels, EEG power density in both frequency ranges significantly increased (EEG alpha activity starting at CT −6; sigma/beta activity starting at CT 4). EEG alpha activity showed peaks which were statistically determined to occur during both evenings (at CT −4 and at CT 20) while EEG sigma/beta activity only showed one significant peak in the second evening (at CT 20). In between both peaks, EEG alpha activity significantly decreased with a trough at CT 3. Compared to CT 20 (i.e. directly prior to WMZ 2; see also below) EEG alpha as well as sigma/beta activity was significantly lower from CT 21 until CT 26 (p < 0.05).

#### Subjective and objective sleepiness changes during the wake maintenance zones

The effect of the WMZ on subjective and objective sleepiness was investigated via the same three analyses which were performed for cognitive performance tests (see Fig. S1). First we compared the absolute levels of sleepiness between the two WMZs (WMZ 1 vs. WMZ 2). Participants were significantly less sleepy during WMZ 1 compared to WMZ 2 (F_1,17_ = 9.495, p = 0.007, d = 1.178), and in the hour prior to WMZ 1, participants were also significantly less sleepy compared to the hour prior to WMZ 2 (PH 1 vs. PH 2; F_1,11_ = 5.388, p = 0.041, d = 1.028; Fig. 2h; Table S2.2.1). During WMZ 1, absolute EEG delta/theta activity (4. 0–5.0 Hz) was significantly lower than during WMZ 2 (F_1,27_ = 21.460, p < 0.001, d = 1.177), while absolute EEG alpha (10–14 Hz) and sigma/beta activity (15.5–23 Hz) showed no significant differences between the WMZs (Fig. 3a). In the hour prior to WMZ 1 compared to the hour prior to WMZ 2 (PH 1 vs. PH 2) there was also significantly lower EEG delta/theta (F_1,19_ = 12.213, p = 0.002, d = 1.484) and sigma/beta activity (F_1,11_ = 8.063, p = 0.017, d = 1.097) while EEG alpha activity showed no significant difference between the hours prior to the WMZs (Table S2.2.1).

We then compared subjective sleepiness and the waking EEG during each WMZ with the preceding hour (WMZ 1 vs. PH 1; and WMZ 2 vs. PH 2). Subjective sleepiness was not significantly reduced during either WMZ. In the wake EEG (0.5–25 Hz) we found significantly lower power density during WMZ 1 compared to the prior hour in the EEG delta/theta frequency range between 3.0–7.0 Hz (F_1,38_ = 4.519, p = 0.040, d = −0.643). During WMZ 2 EEG power density was significantly lower in the delta/theta (4.0–5.0 Hz; F_1,38_ = 11.901, p = 0.001, d = −1.021), alpha (10.0–14.0 Hz; F_1,35_ = 5.900, p = 0.020, d = −0.792) and sigma/beta (15.5–23.0 Hz; F_1,31_ = 7.612, p = 0.010, d = −0.805) frequency ranges compared to the hour prior to WMZ 2 (Fig. 3a,b; Table S2.2.2).

As in the analysis of cognitive performance, we determined whether extended wakefulness had a differential impact on the change in subjective and objective sleepiness between both WMZs (WMZ 1/PH 1 vs. WMZ2/PH 2). The changes between both WMZs showed no difference in subjective sleepiness, but revealed a significantly stronger reduction in EEG power density during WMZ 2 in the high sigma/beta range (17.5–22.5 Hz) when compared to WMZ 1 (F_1,10_ = 6.715, p = 0.027; Fig. 3b; Table S2.2.3).

#### EEG power density reductions during the WMZ coincided with stable subjective sleepiness and improved cognitive performance

The finding that the PVT lapses showed a significantly greater decrease during WMZ 2 and the finding of a larger reduction in EEG sigma/beta activity during WMZ 2 compared to WMZ 1, warranted a closer look at the differences in the time courses between cognitive performance (as assessed in the PVT) and subjective/objective sleepiness during both WMZs. As visually apparent in Fig. 3c–e (the data is expressed relative to the hour preceding each WMZ), PVT lapses, subjective sleepiness (see also Fig. S2) and EEG beta activity remained constant during WMZ 1 whereas EEG delta/theta and alpha activity showed a significant reduction (p < 0.05). During WMZ 2, the EEG reductions were significantly more pronounced and also EEG sigma/beta activity showed a significant reduction while PVT lapses improved (EEG delta/theta activity: F_1,67_ = 11.414, p = 0.001, d = −0.835; alpha activity: F_1,68_ = 2.075, p = 0.035, d = −0.335; sigma/beta activity: F_1,68_ = 11.679, p = 0.001, d = −0.794; PVT lapses: F_1,39_ = 7.766, p = 0.008, d = −1.210; Main effects of WMZ; Table S2.3).

#### Subjective and objective sleepiness after the recovery night

After the recovery night and during polychromatic bright light exposure (see Methods) subjective sleepiness returned to levels that were no longer significantly different from the first morning of the CR (i.e. p > 0.05; Fig. 2h). Also, EEG power density (0.5–25 Hz) returned to the level of the start of the constant routine (Fig. 3a).

### Associations between subjective and objective sleepiness, and cognitive performance

A correlation analysis showed that subjective sleepiness across the 40 h CR protocol was significantly associated with many of the cognitive performance tests. The sleepier participants rated themselves, the worse they performed on the PVT, the Go/No-Go test, the 2-Back Test, the Word-Memory Test, and Negative Affect Test (r ranged from −0.500 to 0.489 and p < 0.032; see Table S3.0 for all exact r and p-values). Subjective sleepiness did not show significant correlations with the Addition Task, the 3-Back Test or the Abstract Reasoning Task.

The EEG power density during the 40 h CR was also associated with subjective sleepiness and cognitive performance. In this analysis, the same frequency bands were used that were previously shown to be affected by the WMZs. An increase in EEG delta/theta activity (4–5 Hz) significantly correlated with greater subjective sleepiness and worse performance on most cognitive tests, except for the 3-Back and Addition Task (r ranged from −0.431 to 0.519; p < 0.018). An increase in EEG alpha activity (10–14 Hz) did not significantly correlate with subjective sleepiness but correlated significantly with worse performance on the PVT, the 2-Back Test and the Negative Affect Test (r ranged from −0.158 to 0.268; p < 0.034). Higher EEG sigma/beta activity (15.5–23 Hz) was significantly associated with an increase in subjective sleepiness and also a worsening of performance on the PVT, Go/No-Go, 2-Back, Word-Pair Memory Test, and the Negative Affect Test (r ranged from −0.322 to 0.328; p < 0.024).

### Sleep stages during the baseline and the recovery night

Sleep during the baseline night was compared to sleep during the recovery night (see also Table S4). Participants had (as expected) significantly shorter sleep onset latency (SOL) and a significantly longer total sleep time (TST) in the recovery night (SOL: F_1,9_ = 8.152, p = 0.018; TST: F_1,9_ = 5.070, p = 0.049). During the recovery night participants had also significantly more deep sleep (stage N3; F_1,12_ = 59.252, p < 0.001) than during the baseline night. This occurred at the cost of significantly less light sleep (stage N1: F_1,12_ = 20.894, p = 0.001; and stage N2: F_1,10_ = 19.271, p = 0.001). Deep sleep latency was also significantly shorter, whereas wakefulness after sleep onset (WASO), sleep efficiency (SE), REM sleep latency and REM sleep showed no significant differences between baseline and recovery night (N3 onset latency: F_1,22_ = 30.753, p < 0.001; WASO: F_1,10_ = 1.266, p = 0.286; SE: F_1,10_ = 1.010, p = 0.338; REM sleep latency: F_1,12_ = 0.802, p = 0.388; REM sleep: F_1,11_ = 0.441, p = 0.520).

## Discussion

During 40 hours of sleep deprivation in a CR protocol, performance of sustained attention (PVT) and response inhibition (Go/No-Go test) showed a circadian modulation with improvements during the WMZs, while performance on higher executive functioning tasks did not. Subjective sleepiness showed a similar time course as sustained attention, but was not significantly reduced during the WMZs. EEG delta/theta and sigma/beta activity increased over time but also demonstrated a circadian influence by revealing a significant power reduction during the WMZs when compared to the preceding hour. This reduction, together with a reduction in EEG alpha activity, was more pronounced in the second WMZ and coincided with stable subjective sleepiness and improved cognitive performance.

As expected and shown in previous studies^16,18^ cognitive performance and sleepiness became worse between the first and the second evening which can be attributed to sleep deprivation effects. We found that the alerting effect during the WMZs was most evident during the second WMZ but this does not necessarily mean that the alerting effect of the first WMZ was much weaker. The hour preceding the WMZ was used as a baseline to compare the effects of the WMZs. In the hour preceding the first WMZ participants were still quite alert since it occurred during the late afternoon on a day when the participants were well rested. Therefore, the alerting effect during the first WMZ might seem smaller due to a ceiling effect.

We observed the clearest alerting effect of both WMZs in objective measures (EEG activity) and in the less difficult cognitive tasks. The absence of a WMZ effect on executive functioning could be explained by a lower susceptibility to sleep deprivation or mechanisms to compensate for greater sleepiness. A previous study found that negative effects of sleep deprivation on executive functioning could be compensated for by stronger activation of cerebral responses promoting task specific attention, or by activation of cerebral responses that were not active when the task was performed in well rested conditions^27^. Also, all our participants had to practice all the tests on the day preceding the 40 h constant routine in order to familiarize themselves with the cognitive tasks. Yet, any learning effects during the CR cannot fully be ruled out, even though we used different stimuli which were randomly presented for each task. We did not ask for the strategies subjects used to master the task. Therefore, it might be that the strategy to complete a task may have changed during the CR and thus, task performance could be, at least in part, the result of changes in task strategy.

Most previous studies report improvements during the WMZ in sustained attention tests, and some also showed improvements in memory tests, as well as lower subjective sleepiness, while others found no changes during the WMZ^15–24^. We found improvements during the WMZs in sustained attention and response inhibition when we compared the WMZs to the preceding hour. These results are similar to a previous study, where improvements in the visual version of the PVT and the digit symbol substitution test were found during the WMZs on both evenings of a 50 h CR^16^. That same study also reported better performance for an auditory version of the PVT and for subjective sleepiness only during the WMZ of the second evening^16^. We also found an improvement in the auditory PVT only during the WMZ of the second evening, but no significant reduction of subjective sleepiness during both WMZs. Although in the evenings the performance decline in sustained attention as well as the augmentation of subjective sleepiness became steepest directly after DLMO (see Fig. S2). Thus, in our participants the circadian arousal signal during the WMZs may still be strong enough to ‘counteract’ homeostatic sleep pressure, since it kept subjective sleepiness stable until the ‘sleep gate’ (as the time around DLMO has been called^9^) was opened.

Low frequency EEG activity has been shown to correlate with worse cognitive performance and more lapses in sustained attention during extended wake episodes, which makes it a useful measure of objective sleepiness^3,18,28^. We also found that higher EEG delta/theta activity correlated with higher subjective sleepiness and a decline in most cognitive tests. In the EEG delta/theta range, which is known to be mainly under homeostatic control, we found a lower activity during the first WMZ and an increase over time leading to significant higher absolute EEG power during the second WMZ. Our results are in agreement with a model describing the time course of sleep propensity by a multiplicative interaction of two sleep drives, a homeostatic and a circadian one^29,30^. This model would correctly predict the presence of both WMZs, and in addition would predict the sleep propensity in WMZ 2 to be higher than in WMZ 1 if the circadian drive for sleep never attains zero values^29^.

Our results showed a decrease of EEG activity in low (delta/theta) and higher (alpha, sigma/beta) frequency ranges during the WMZs when compared to the preceding hour. EEG power in these frequency ranges also demonstrated a significant modulation over time during the 40 h CR, as has been shown by others^31–33^. The potential influence of the circadian arousal signal specifically during the WMZ might be illustrated by the differential decline in EEG activity when we compared both WMZs. The reduction in the EEG sigma/beta frequency range was significantly stronger during the second compared to the first WMZ, suggesting that EEG power density changes in this frequency range are contributing to modulations of the circadian arousal signal, depending on prior duration of wakefulness.

We could confirm the circadian modulation of the time course of EEG alpha activity as shown by others^32^. The peak of EEG alpha activity on both days was in the afternoon just prior to the start of the WMZs. While subjective sleepiness remained stable and cognitive performance remained stable or was even improved during the WMZs, EEG alpha activity declined during the WMZ. This is interesting since the neurobehavioral substrate of the circadian modulation of alertness and cognitive performance is still not fully understood. A recent study by Muto and colleagues showed for the first time that functional MRI responses during sustained attention tests demonstrated a clear circadian modulation (especially in subcortical areas like the midbrain, cerebellum, basal ganglia, and thalamus)^34^. They also showed that this circadian modulation was closely related to the melatonin secretion pattern, with increased cortical responses during the WMZ immediately before the DLMO. Another study showed that during the WMZ, the postero-lateral hypothalamus is responsible for integration of the homeostatic sleep pressure and the circadian modulation of cognitive performance^25^. These studies are beginning to elucidate the underlying mechanisms of the brain that give rise to the circadian modulation of cognitive performance. Our findings of differential changes of EEG activity during the WMZs, depending on prior duration of wakefulness, may add to this understanding.

The reason why we found a WMZ effect in objective but not in subjective sleepiness could also be because of dissociation between the two, likely caused by a differential perception of subjective sleepiness. And it indicates a subjective adaptation to sleep depth as was shown in studies with chronic sleep restriction^35^. While many studies have, other studies have not, found correlations between subjective and objective sleepiness, and it has been hypothesized that the two reflect different physiological mechanisms underlying sleepiness^36–38^. We did find correlations in the time course over the 40 h CR between subjective sleepiness and delta/theta or sigma/beta EEG activity (see Table S3). The small effect size for subjective sleepiness indicates that our (small) sample size may have led to low statistical power for subjective measures, while the statistical power for objective sleepiness measures was large enough to reveal significant differences, as shown by the large effect sizes. Thus, the differences between subjective and objective sleepiness measures might also reflect the discrepancy in statistical power between the two variables, as shown by the different effect sizes.

We are aware that by analyzing the data in “three different ways” (see Results) we used parts of the same data for three different comparisons. But, since the comparisons of absolute and relative data are based on different a priori assumptions and were used in separate regression models, we refrained from using an a priori Bonferroni adjustment. Nevertheless, p-values within each model were adjusted for multiple comparisons by using the LSD method (see Methods).

We included only healthy young male participants. Perhaps we would have seen more of a WMZ alerting effect if we had included female participants since a recent study showed a stronger circadian rhythm in cognitive performance among women^23^. Including other age groups would also be important. One reason why we only included males was that the study was part of a larger project with genetic samples where the group was required to have as little hormonal and other variability as possible.

Our results may be of particular interest concerning shift work. Especially during night shifts, there is a misalignment between the sleep-wake cycle and the circadian rhythm of alertness and cognitive performance. Taking the timing of the WMZ into account could be useful for example when timing naps. On the other hand, late chronotypes may benefit especially from the WMZ and experience fewer problems with night shifts if their WMZ overlaps with at least part of the night shift.

After the recovery night, performance on all cognitive tests returned to baseline levels (start of CR). In fact, the reaction times of the PVT were even faster than at the start of the CR when the participants were well rested. This contradicts previous findings that one 8 h recovery night is insufficient for complete cognitive recovery after acute sleep deprivation^39–42^. However, our setting was different because our study included a bright light exposure in the morning after the recovery night which may have aided the full cognitive recovery.

To summarize, we found that after 37.5 hours of extended wakefulness (WMZ 2) the circadian influence on the EEG delta/theta, alpha and sigma/beta ranges was stronger than under conditions of ‘normal’ sleep pressure (i.e. WMZ 1). Also in cognitive performance, the improvements during the second WMZ were more pronounced than during the first WMZ. The decrease in EEG activity during the WMZs occurred while subjective sleepiness remained stable and cognitive performance either remained stable or even improved. The differential changes in EEG power and cognitive performance during the WMZs reflect that the circadian arousal signal is modulated by the duration of prior wakefulness.

## Methods

### Participants

Participants were recruited via flyers at local universities. Twelve male participants were included in the study (age: 25.3 ± 2.6 yrs; mean ± SD). All participants completed a medical screening, an interview and filled out five screening questionnaires. To be included the participants had to be healthy and without any psychiatric or sleep disorders. They were only included if they were not taking any medications and if they were non-smokers. The five screening questionnaires were: a general entrance questionnaire, the Pittsburgh Sleep Questionnaire Index^43^ (3.4 ± 0.9; mean ± SD), the Morningness-Eveningness Questionnaire^44^ (50.3 ± 7.4; mean ± SD), the Munich Chronotype Questionnaire^45^ (4.5 ± 0.6; mean ± SD) and the Seasonal Pattern Assessment Questionnaire^46^ (7.1 ± 2.6; mean ± SD). Other inclusion criteria were no night shift work during the last eight weeks and no travel to other time zones in the last three months. The first night in the laboratory served as adaptation night and was polysomnographically recorded. None of the participants had periodic leg movements (PLM; score with arousals; cutoff < 10/h), or a sleep disorder (cutoff: apnoea/hypopnea index < 15/h). All participants gave a written informed consent and the study was approved by the local ethical committee of the Charité University Medicine Berlin (Germany) and conformed to the tenets of the Declaration of Helsinki.

### Study design

Participants kept their regular habitual bedtimes for one week preceding the study (controlled by actigraphy and sleep diaries; habitual bedtime 23:50 ± 0:43; habitual wake time 7:51 ± 0:43; mean ± SD). On day 1 each participant came to the laboratory in the evening 3 h prior to habitual bedtime (Fig. 1). No more than one participant visited the laboratory at the same time. The first night was an adaptation night of approximately 8 h. On day 2 the participants spend the day in the laboratory with room lighting (LED ceiling lights; ≈500 lx in a vertical direction at eye level; 2800 K; 1.85 W/m^2^). In the evening of day 2, six hours prior to habitual bedtime, the participants stayed in dim light (<5 lx) and hourly saliva samples were collected. The dim light was produced by a standing luminaire with a halogen bulb (polychromatic white light). The standing luminaire was adjusted to be lower than 5 lx (in a vertical direction anywhere in the room) as measured by a luxmeter (Showtec, Digital Luxmeter) and was kept at this illuminance level on the evening of day 2 and throughout the constant routine. The saliva samples were assayed for melatonin concentrations and the timing of the onset of melatonin concentrations in dim light (DLMO) was used as the circadian phase marker. This was followed by a baseline night (8 h) after which the 40 h CR protocol started. During the CR participants remained in dim light (<5 lx), stayed in bed in a semi-recumbent position (~45°) at all times, and received iso-caloric snack opportunities every hour (150 kcal). The laptop screen used during the cognitive tests was maximally dimmed so that illuminance measured at eye level in a vertical angle of gaze was below 5 lx at all times. The participants had no information about the time of day and the use of cell-phones or tablets was not allowed. Saliva samples were collected hourly (see supplement), core body temperature (rectal probe; data not reported here) and the electroencephalogram were recorded continuously. The 40 h CR protocol was followed by an 8 h recovery night. On day 5, one hour after wake-up time participants were exposed to 3 h of polychromatic bright white light (1.300 lx; measured at eye level in a vertical angle of gaze) for purposes not reported here.

### Cognitive tests and subjective sleepiness

Two cognitive test batteries with a total of 7 different tasks were performed during the CR alternating every two hours (on a laptop with dimmed screen). The first battery consisted of the N-Back Tests (with three levels: 0-Back, 2-Back, 3-Back Test)^47^, a 5 min version of the auditory Psychomotor Vigilance Task (PVT)^48^, a 3 min visual Addition Task^49^, and a task where participants had to recognize negative emotions (Negative Affect)^50^. The second cognitive test battery consisted of a visual 3 min Go/No-Go task^51^, a delayed recall Word-Pair Memory Task^52^ and an Abstract Reasoning Task^53^. The Karolinska Sleepiness Scale (KSS)^54^ was included in both batteries and was performed every hour at the beginning of the cognitive testing. For visual illustration, results were averaged every 1–2 hours for the entire study protocol and the morning following recovery sleep.

### Wake-EEG and sleep recordings

Every hour, following the cognitive tests, participants performed the Karolinska Drowsiness Test (KDT; 3 min)^31^. They had to keep their eyes open, and refrain from moving, as well as blink as few times as possible. The recordings were performed with six EEG derivations (F3, F4, C3, C4, O1 and O2), referenced against mastoids (A1 and A2) using a Rembrandt system (Monet 24-CPU hardware, TMS International, Enschede, The Netherlands; and Rembrandt 7.5 software, Medcare Automation, Amsterdam, The Netherlands). The sampling rate of the EEG was 160 Hz and recordings were low-pass filtered (70 Hz) and high-pass filtered (0.3 Hz). After study completion, the EEG MATLAB toolbox (The MathWorks, Inc., Natick, Massachusetts, United States) was used for manual artifact removal (movements and blinking) and spectral analysis. Spectral analysis was performed by applying a Fast Fourier Transformation in the range between 0.5 to 80.5 Hz with a resolution of 0.5 Hz. Here, we report data in the frequency range between 0.5 and 25 Hz from a frontal derivation (F4). Recordings from two out of the twelve subjects could not be included in the wake-EEG analysis due to technical problems.

Polysomnographic sleep recordings of the baseline and recovery night were visually scored in compliance with the Guidelines of the American Academy of Sleep Medicine (2007)^55^. The recovery night of one participant was excluded from the analysis due to technical problems during the recording.

### Dim light melatonin onset

Dim light melatonin onset (DLMO) was used in order to assess individual circadian phase, determined by the software tool of Danilenko *et al*.^56^. The DLMO was defined as the time when the melatonin concentration exceeded two standard deviations (2 SD) after three low daytime time points^57^. See supplemental text for inter- and intra-assay values (page 17).

### Timing of the wake maintenance zone

The WMZ was defined as the time range 3 h prior to DLMO on the first evening of the CR (i.e. DLMO = CT 0 which was on average at 21:17 ± 1:09 h; SD; range = 19:13 until 22:36). WMZ 1 was on the first day between 10.5 and 13.5 h after wake time and WMZ 2 on the second day between 34.5 and 37.5 h being awake (±0.9 h; SD).

### Statistical analysis

All statistics were performed in IBM SPSS Statistics for Windows, Version 23.0. (IBM Corp., Armonk, N.Y., USA). Cognitive performance, subjective sleepiness and EEG power densities were first expressed relative to the DLMO of the first CR evening (=CT 0). For cognitive performance, mixed linear models with fixed factor “TIME” and random factor “PARTICIPANT” were performed on log_10_-transformed data to analyze differences across the 40 h CR and to compare cognitive performance after the recovery night with the start of the CR. In a second step, the effects of the WMZ were compared to the preceding hour by mixed linear models with the fixed factors “TIME POINT” (which was either “during WMZ” or “hour prior to WMZ”) and “DAY” (1^st^ biological day or 2^nd^ biological day) and again the random factor “PARTICIPANT”. The same mixed linear models were performed on the log_10_-transformed EEG power densities of the KDTs in the wake-EEG for every 0.5 Hz frequency bin in the range from 0.5 to 25 Hz. In the analysis of the decline in EEG frequency ranges during the WMZs, mixed linear models were performed with the fixed factors “TIME” (= time points around the WMZs) and “WMZ” (WMZ 1 or WMZ 2). All post-hoc tests were corrected for multiple comparisons by the Least Significance Test (LSD). Correlation analysis was performed by using a Spearman’s Rho correlation for hourly bins of cognitive performance, subjective sleepiness and EEG power bins in three different frequency ranges.

## Electronic supplementary material

Supplemental Material
