# Supplementary material for: The alerting effect of the wake maintenance zone during 40 hours of sleep deprivation
Source: Sci Rep. 2018 Jul 20;8:11012. doi: 10.1038/s41598-018-29380-z (PMC6054682; doi:10.1038/s41598-018-29380-z)
Supplement: Supplementary file 1 — Supplemental Material [file 41598_2018_29380_MOESM1_ESM.pdf]

Supplemental Material

The alerting effect of the wake maintenance zone during 40 hours of sleep deprivation

Jan de Zeeuw[1,4], Sophia Wisniewski[1,3], Alexandra Papakonstantinou[1,3], Frederik Bes[1,3], Amely Wahnschaffe[1,3], Mandy Zaleska[4], Dieter Kunz[1,3,4#], Mirjam Münch[1,2,3#*]

[1]Charité – Universitätsmedizin Berlin, corporate member of Freie Universität Berlin, Humboldt-Universität zu Berlin, and Berlin Institute of Health; Institute of Physiology, Sleep Research & Clinical Chronobiology; [2]Charité – Universitätsmedizin Berlin, corporate member of Freie Universität Berlin, Humboldt-Universität zu Berlin, and Berlin Institute of Health, Institute of Medical Immunology, Laboratory of Chronobiology; [3]Clinic for Sleep & Chronomedicine, St. Hedwig-Krankenhaus, Berlin; [4]Intellux GmbH, Berlin, Germany; [#]co-last authors

*Corresponding author:
Mirjam Münch, PhD
Charité Universitätsmedizin Berlin
Institute of Physiology
Sleep Research & Clinical Chronobiology
c/o St. Hedwig-Krankenhaus
Grosse Hamburger Strasse 5-11
10115 Berlin
Phone: 0049 2311 2901
Fax: 0049 2311 2903
Email: mirjam.muench@charite.de

Cognitive Tests	Main Effect of TIME
PVT Median Reaction Times	$F_{1,20} = 4.042$, $p < 0.001$
PVT Lapses	$F_{1,20} = 3.208$, $p < 0.001$
Go/No-Go	$F_{1,20} = 4.815$, $p < 0.001$
2-Back	$F_{1,20} = 3.519$, $p < 0.001$
3-Back	ns
Word-Memory	ns
Addition Task	$F_{1,20} = 3.611$, $p < 0.001$
Abstract Reasoning	ns
Negative Affect	$F_{1,20} = 2.810$; $p < 0.001$

Table S1.1. Cognitive performance across 40 hours. The first column shows: PVT median reaction times in milliseconds; number of PVT lapses; % correct on the Go/No-Go; % correct on the 2-Back test; % correct on the 3-Back Test; % correct on the Word-Memory Test; Efficiency on the Addition Task (% correct / Reaction Time); % correct on the Abstract Reasoning Test; % correct on Negative Affect. The second column shows the F- and p-values for the statistical variation of the time course of cognitive performance tests across 40 h of extended wakefulness (= main effect of TIME); ns = not significant.

Cognitive Tests	WMZ 1 vs. WMZ 2				Hour before WMZ 1 vs. Hour before WMZ 2			
	F- and p-values	Effect Size (d)	WMZ 1 Mean (± SD)	WMZ 2 Mean (± SD)	F- and p-values	Effect Size (d)	Hour before WMZ 1 Mean (± SD)	Hour before WMZ 2 Mean (± SD)
PVT Median Reaction Times	ns	0.293	271.3 (24.2)	279.0 (28.4)	ns	0.329	270.9 (20.5)	279.1 (29.0)
PVT Lapses	$p = 0.047$	0.968	0.3 (0.7)	2.2 (2.7)	$p = 0.008$	1.108	0.5 (0.9)	5.5 (6.3)
Go/No-Go	$F_{1,11} = 7.084$ $p = 0.022$	-0.747	94.5 (4.9)	87.9 (11.5)	$F_{1,10} = 6.774$ $p = 0.027$	-0.697	92.3 (6.6)	85.1 (12.9)
2-Back	$F_{1,16} = 13.921$ $p = 0.002$	-1.160	97.7 (2.3)	92.4 (6.0)	ns	-0.623	95.5 (5.2)	91.8 (6.4)
3-Back	ns	-0.501	87.0 (5.8)	82.4 (11.6)	$F_{1,13} = 8.180$ $p = 0.014$	-0.471	85.2 (10.2)	79.7 (12.9)
Word-Memory	ns	-0.094	90.0 (16.5)	88.3 (18.9)	$F_{1,10} = 10.495$ $p = 0.021$	-0.804	95.0 (8.0)	85.0 (5.7)
Addition Task	ns	-0.185	0.020 (0.007)	0.019 (0.008)	$F_{1,11} = 10.729$ $p = 0.007$	-1.135	0.023 (0.004)	0.017 (0.006)
Abstract Reasoning	ns	-0.263	58.1 (27.8)	51.9 (18.6)	ns	-0.336	61.4 (21.6)	51.9 (18.0)
Negative Affect	ns	-0.646	74.2 (12.4)	66.2 (12.4)	ns	-0.885	74.3 (9.9)	66.0 (8.9)

Table S1.2.1. Cognitive performance: comparison between WMZs and between prior hours. The first column of the table lists the cognitive performance tests. The second column shows the F- and p-values of the comparison between WMZ 1 and WMZ 2. The third column shows the effect size (Cohen's d). The fourth and fifth columns show the means (± SD). The sixth column shows the F- and p-values of the comparison between the hour before WMZ 1 and the hour before WMZ 2. The seventh column shows the effect size (Cohen's d). The eighth and ninth columns show the means (± SD). Since PVT lapses were not normally distributed they were analyzed with the Wilcoxon Test. The cognitive performance tests are presented as: PVT median reaction times in milliseconds; number of PVT lapses; % correct on the Go/No-Go; % correct on the 2-Back test; % correct on the 3-Back test; % correct on the Word-Memory test; Efficiency on the Addition Task (% correct / reaction time); % correct on the Abstract Reasoning Test; % correct on Negative Affect; ns = not significant.

Cognitive Tests	Preceding Hour vs. WMZ 1				Preceding Hour vs. WMZ 2			
	F- and p-values	Effect Size (d)	Hour prior to WMZ 1 Mean (± SD)	WMZ 1 Mean (± SD)	F- and p-values	Effect Size (d)	Hour prior to WMZ 2 Mean (± SD)	WMZ 2 Mean (± SD)
PVT Median Reaction Times	ns	0.018	270.9 (20.5)	271.3 (24.2)	ns	-0.004	279.1 (29.0)	279.0 (28.4)
PVT Lapses	ns	-0.289	0.5 (0.9)	0.3 (0.7)	p = 0.012	-0.687	5.5 (6.3)	2.2 (2.7)
Go/No-Go	$F_{1,12}$ = 5.142 p = 0.043	0.380	92.3 (6.6)	94.5 (4.9)	ns	0.227	85.1 (12.9)	87.9 (11.5)
2-Back	ns	0.550	95.5 (5.2)	97.7 (2.3)	ns	0.098	91.8 (6.4)	92.4 (6.0)
3-Back	ns	0.224	85.2 (10.2)	87.0 (5.8)	ns	0.223	79.7 (12.9)	82.4 (11.6)
Word-Memory	ns	-0.386	95.0 (8.0)	90.0 (16.5)	ns	0.191	85.0 (5.7)	88.3 (18.9)
Addition Task	ns	-0.501	0.023 (0.004)	0.020 (0.007)	ns	0.253	0.017 (0.006)	0.019 (0.008)
Abstract Reasoning	ns	-0.131	61.4 (21.6)	58.1 (27.8)	ns	-0.154	51.9 (18.0)	51.9 (18.6)
Negative Affect	ns	0.013	74.3 (9.9)	74.2 (12.4)	ns	0.015	66.0 (8.9)	66.2 (12.4)

Table S1.2.2. Cognitive performance: comparisons of each WMZ with its preceding hour. The first column of the table lists the cognitive performance tests. The second column shows the F- and p-values of the comparison between WMZ 1 and its preceding hour. The third column shows the effect size (Cohen's d; see also Fig. S3). The fourth and fifth columns show the means (± SD). The sixth column shows the F- and p- values of the comparison between WMZ 2 and its preceding hour. The seventh column shows the effect size (Cohen's d). The eighth and ninth columns show the means (± SD). Since PVT lapses were not normally distributed they were analyzed with the Wilcoxon Test. The cognitive performance tests are presented as: PVT median reaction times in ms; number of PVT lapses; % correct on the Go/No-Go; % correct on the 2-Back test; % correct on the 3-Back test; % correct on the Word-Memory test; Efficiency on the Addition Task (% correct / reaction time); % correct on the Abstract Reasoning Test; % correct on Negative Affect; ns = not significant.

Cognitive Tests	Change in WMZ 1 vs. Change in WMZ 2			
	F- and p-values	Effect Size (d)	WMZ 1 Mean (± SD)	WMZ 2 Mean (± SD)
PVT Median Reaction Times	ns	0.057	1.0 (0.04)	1.0 (0.04)
PVT Lapses	p = 0.009	-0.986	-0.3 (1.1)	-3.3 (4.3)
Go/No-Go	ns	0.207	1.03 (0.05)	1.04 (0.08)
2-Back	ns	-0.119	1.02 (0.06)	1.01 (0.06)
3-Back	ns	0.228	1.02 (0.11)	1.05 (0.16)
Word-Memory	ns	0.491	0.95 (0.17)	1.06 (0.26)
Addition Task	$F_{1,11}$ = 6.843 p = 0.025	0.808	0.9 (0.3)	1.1 (0.2)
Abstract Reasoning	ns	0.047	1.06 (0.72)	1.09 (0.48)
Negative Affect	ns	-0.012	1.01 (0.16)	1.01 (0.19)

Table S1.2.3. Cognitive performance: comparisons of changes between wake maintenance zones. The first column of the table lists the cognitive performance tests. The second column shows the F- and p-values for the comparison of the changes during WMZ 1 and WMZ 2. The third column shows the effect size (Cohen's d). The fourth and fifth columns show the means (± SD). For PVT lapses the Wilcoxon test was performed. The cognitive performance tests are presented as relative values (each WMZ relative to its preceding hour); ns = not significant.

Subjective Sleepiness/EEG Activity	Main Effect of TIME
Subjective Sleepiness	$F_{1,41}$ = 8.596, p < 0.001
EEG delta/theta activity (4 - 5 Hz)	$F_{41,232}$ = 6.812, p < 0.001
EEG alpha activity (10 - 14 Hz)	$F_{41,208}$ = 2.861, p < 0.001
EEG sigma/beta activity (15.5 - 23 Hz)	$F_{41,213}$ = 2.607, p < 0.001

Table S2.1. Subjective and objective sleepiness across 40 hours. F- and p-values for the time course of subjective sleepiness and EEG frequency ranges across 40 h of extended wakefulness (= main effect of TIME).

Subjective Sleepiness/EEG Activity	WMZ 1 vs. WMZ 2		Hour before WMZ 1 vs. Hour before WMZ 2	
	F- and p-values	Effect Size (d)	F- and p-values	Effect Size (d)
Subjective Sleepiness	$F_{1,17} = 9.495$ $p = 0.007$	1.178	$F_{1,11} = 5.388$ $p = 0.041$	1.028
EEG delta/theta activity (4 - 5 Hz)	$F_{1,27} = 21.460$ $p < 0.001$	1.177	$F_{1,19} = 12.213$ $p = 0.002$	1.484
EEG alpha activity (10 - 14 Hz)	ns	0.242	ns	0.744
EEG sigma/beta activity (15.5 - 23 Hz)	ns	0.373	$F_{1,11} = 8.063$ $p = 0.017$	1.097

Table S2.2.1. Subjective and objective sleepiness: comparison between WMZs and between prior hours. The first column of the table lists the subjective sleepiness and EEG activity ranges. The second column shows the F- and p-values for the comparison between WMZ 1 and WMZ 2 with the effect size in the third column (Cohen's d). The fourth column shows the F- and p-values of the comparison between the hour prior to WMZ 1 and the hour prior to WMZ 2, with the effect size in the fifth column (Cohen's d); ns = not significant.

Subjective Sleepiness/EEG Activity	Preceding Hour vs. WMZ 1		Preceding Hour vs. WMZ 2	
	F- and p-values	Effect Size (d)	F- and p-values	Effect Size (d)
Subjective Sleepiness	ns	0.045	ns	-0.123
EEG delta/theta activity (3 - 7 Hz)	$F_{1,37}$ = 8.762, p = 0.005	-0.850	ns	-1.037
EEG delta activity (4 - 5 Hz)	$F_{1,38}$ = 4.519, p = 0.040	-0.643	$F_{1,38}$ = 11.901, p = 0.001	-1.021
EEG alpha activity (10 - 14 Hz)	ns	-0.443	$F_{1,35}$ = 5.900, p = 0.020	-0.792
EEG sigma/beta activity (15.5 - 23 Hz)	ns	-0.058	$F_{1,31}$ = 7.612, p = 0.010	-0.805

Table S2.2.2. Subjective and objective sleepiness: comparisons of each WMZ with its preceding hour. The first column of the table lists the subjective sleepiness and EEG activity ranges. The second column shows the F- and p-values for the comparison between WMZ 1 and the preceding hour, with the effect size in the third column (Cohen’s d). The fourth column shows the F- and p-values of the comparison between WMZ 2 and the preceding hour, with the effect size in the fifth column (Cohen’s d); ns = not significant.

Subjective Sleepiness/EEG Activity	Change in WMZ 1 vs. Change in WMZ 2
Subjective Sleepiness	ns
EEG sigma/beta activity (17.5 - 22.5 Hz)	$F_{1,10} = 6.715$, $p = 0.027$

Table S2.2.3. Subjective and objective sleepiness: comparisons of changes between wake maintenance zones. The first column of the table lists the subjective sleepiness and EEG activity ranges. The second column shows the F- and p-values for the comparison of the changes during WMZ 1 and WMZ 2; ns = not significant.

	Main Effect of WMZ	Main Effect of TIME
PVT Lapses	$F_{1,39}$ = 7.766, p = 0.008	$F_{1,39}$ = 10.134, p = 0.003
Subjective Sleepiness	ns	ns
EEG delta/theta activity (4 - 5 Hz)	$F_{1,67}$ = 11.414, p = 0.001	$F_{3,63}$ = 2.865, p = 0.044
EEG alpha activity (10 - 14 Hz)	$F_{1,68}$ = 2.075, p = 0.035	$F_{3,65}$ = 4.635, p = 0.005
EEG sigma/beta activity (15.5 - 23 Hz)	$F_{1,68}$ = 11.679, p = 0.001	$F_{3,66}$ = 1.571, p = 0.034

Table S2.3. EEG power density reductions during the WMZ coincided with stable subjective sleepiness and improved cognitive performance. The first column lists the cognitive performance, subjective sleepiness and EEG activity ranges. The second column shows whether the magnitude of the decline during the WMZs was different between WMZ 1 compared to WMZ 2 (main effect of WMZ). The third column shows whether there was a significant decline over time during the WMZ (compared to the start of the WMZ; main effect of TIME). Only PVT lapses showed a significant interaction ($F_{1,39}$ = 7.766, p = 0.008); ns = not significant.

**Subjective Sleepiness vs.:**	**r**	**p-values**
PVT Median Reaction Times	r = 0.430	p < 0.001
PVT Lapses	r = 0.479	p < 0.001
Go/No-Go	r = -0.500	p < 0.001
2-Back	r = -0.250	p < 0.001
3-Back	r = -0.106	ns
Word-Memory	r = -0.259	p < 0.001
Addition Task	r = 0.002	ns
Abstract Reasoning	r = -0.121	ns
Negative Affect	r = -0.142	p = 0.031
**Delta/Theta Activity (4 - 5 Hz) vs.:**	**r**	**p-values**
Subjective Sleepiness	r = 0.506	p < 0.001
PVT Median Reaction Times	r = 0.455	p < 0.001
PVT Lapses	r = 0.519	p < 0.001
Go/No-Go	r = -0.431	p < 0.001
2-Back	r = -0.304	p < 0.001
3-Back	r = -0.143	ns
Word-Memory	r = -0.277	p < 0.001
Addition Task	r = -0.044	ns
Abstract Reasoning	r = -0.175	p = 0.017
Negative Affect	r = -0.309	p < 0.001
**Alpha Activity (10 - 14 Hz) vs.:**	**r**	**p-values**
Subjective Sleepiness	r = 0.060	ns
PVT Median Reaction Times	r = 0.167	p = 0.023
PVT Lapses	r = 0.232	p = 0.002
Go/No-Go	r = 0.015	ns
2-Back	r = 0.268	p = 0.001
3-Back	r = -0.057	ns
Word-Memory	r = -0.128	ns
Addition Task	r = -0.053	ns
Abstract Reasoning	r = 0.052	ns
Negative Affect	r = -0.158	p = 0.033
**Sigma/Beta Activity (15.5 - 23 Hz) vs.:**	**r**	**p-values**
Subjective Sleepiness	r = 0.257	p < 0.001
PVT Median Reaction Times	r = 0.328	p < 0.001
PVT Lapses	r = 0.326	p < 0.001
Go/No-Go	r = -0.205	p = 0.005
2-Back	r = -0.184	p = 0.018
3-Back	r = -0.060	ns
Word-Memory	r = -0.322	p < 0.001
Addition Task	r = 0.006	ns
Abstract Reasoning	r = -0.055	ns
Negative Affect	r = -0.167	p = 0.023

Table S3.0. Associations between subjective and objective sleepiness, and cognitive performance. A correlation analysis was performed on the time course over the 40 h CR. The r and p-values of the correlations between subjective sleepiness and cognitive performance as well as the correlations of EEG frequency ranges with subjective sleepiness and cognitive performance are shown. Correlations were performed with Spearman´s Rho since the data was not normally distributed; ns = not significant.

	Baseline Night Mean (± SD)	Recovery Night Mean (± SD)	F- and p-values
Time in Bed (TIB; min)	478.3 (9.1)	481.9 (4.2)	ns
Total Sleep Time (TST; min)	441.5 (42.6)	465.1 (17.8)	$F_{1,9} = 5.070$, $p = 0.049$
Sleep Onset Latency (SOL; min)	11.0 (9.6)	4.9 (2.5)	$F_{1,9} = 8.152$, $p = 0.018$
REM Sleep Onset Latency (min)	71.7 (30.6)	81.3 (34.6)	ns
N3 Onset Latency (min)	8.9 (3.6)	2.8 (1.9)	$F_{1,22} = 30.753$, $p < 0.001$
Stage N1 (min)	35.8 (10.0)	22.2 (9.3)	$F_{1,12} = 20.894$, $p = 0.001$
Stage N2 (min)	192.2 (27.9)	174.4 (22.1)	$F_{1,10} = 19.271$, $p = 0.001$
Stage N3 (min)	113.2 (29.8)	167.7 (33.3)	$F_{1,12} = 59.252$, $p < 0.001$
REM Sleep (min)	100.4 (28.4)	100.8 (22.5)	ns
NREM (S2+S3)	305.4 (31.6)	342.1 (24.6)	$F_{1,11} = 13.191$, $p = 0.004$
WASO (min)	25.6 (35.8)	12.3 (16.2)	ns
Sleep Efficiency (SE; %)	92.3 (8.7)	96.5 (3.7)	ns

Table S4. Polysomnographic sleep recordings. The first column lists the sleep variables with duration in minutes or %. The second column shows the values for the baseline night; mean (± SD). The third column shows the values for the recovery night; mean (± SD). The fourth column shows the significant F- and p-values. Abbreviations: Time in bed (TIB); Total sleep time (TST, sum of stages N1-3 and REM sleep); Sleep onset latency (SOL); REM sleep onset latency; N3 (deep sleep) onset latency; Stage N1; Stage N2; Stage N3; rapid eye movement (REM) sleep; non-REM (NREM) sleep; WASO = wake after sleep onset; Sleep efficiency (SE; TST / TIB *100).

Supplemental Figures

1. PH 1 ⟷ PH 2
   WMZ 1 ⟷ WMZ 2

2. WMZ 1 ⟷ PH 1
   WMZ 2 ⟷ PH 2

3. $\frac{\text{WMZ 1}}{\text{PH 1}} \longleftrightarrow \frac{\text{WMZ 2}}{\text{PH 2}}$

Figure S1. Statistical comparisons of the Wake Maintenance Zone. This figure gives an overview of the three ways in which the effects of the WMZ on cognitive performance and EEG activity were analyzed. (1.) First, we compared the absolute values between both WMZs and between the previous hours (PH). (2.) Secondly, each WMZ was compared to its respective previous hour. (3.) Finally, each WMZ was expressed relative to its previous hour after which the changes were compared between the two WMZs.

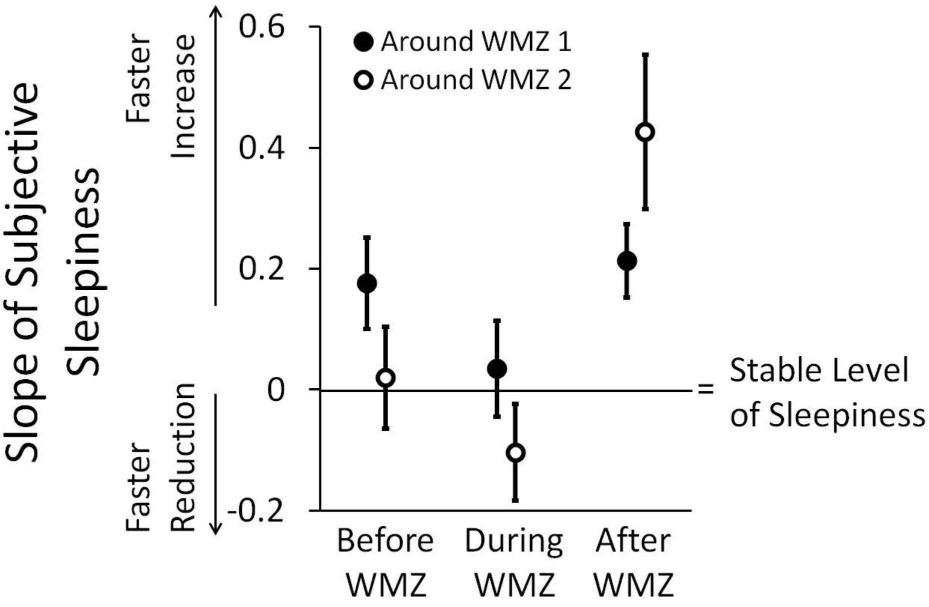

Figure S2. Slope of Subjective Sleepiness. Depicted are the slopes of the time course of subjective sleepiness during the 3 h before each WMZ (WMZ 1: CT -6 until CT -3; WMZ 2: CT 18 until CT 21), during the 3 h of each WMZ (WMZ 1: CT -3 until CT 0; WMZ 2: CT 21 until CT 24), and after each WMZ (WMZ 1: CT 0 until CT 2; WMZ 2: CT 24 until end of CR). The y-axis shows the magnitude of the slope of subjective sleepiness on z-transformed values (i.e. the speed of the increase/reduction of sleepiness). A positive slope indicates an increase of sleepiness and a negative slope indicates a reduction of sleepiness. A mixed model analysis showed a significant main effect for a difference between the three time windows ($F_{2,36} = 8.550$, $p = 0.001$) with a significantly steeper slope (i.e. greater increase of sleepiness) after the WMZs compared to the time before and during both WMZs. There was also a significant interaction between 'time window' and 'biological day' ($F_{2,36} = 3.497$, $p = 0.041$) with the post hoc showing a trend for a larger slope after WMZ 2 than after WMZ 1 ($p = 0.066$).

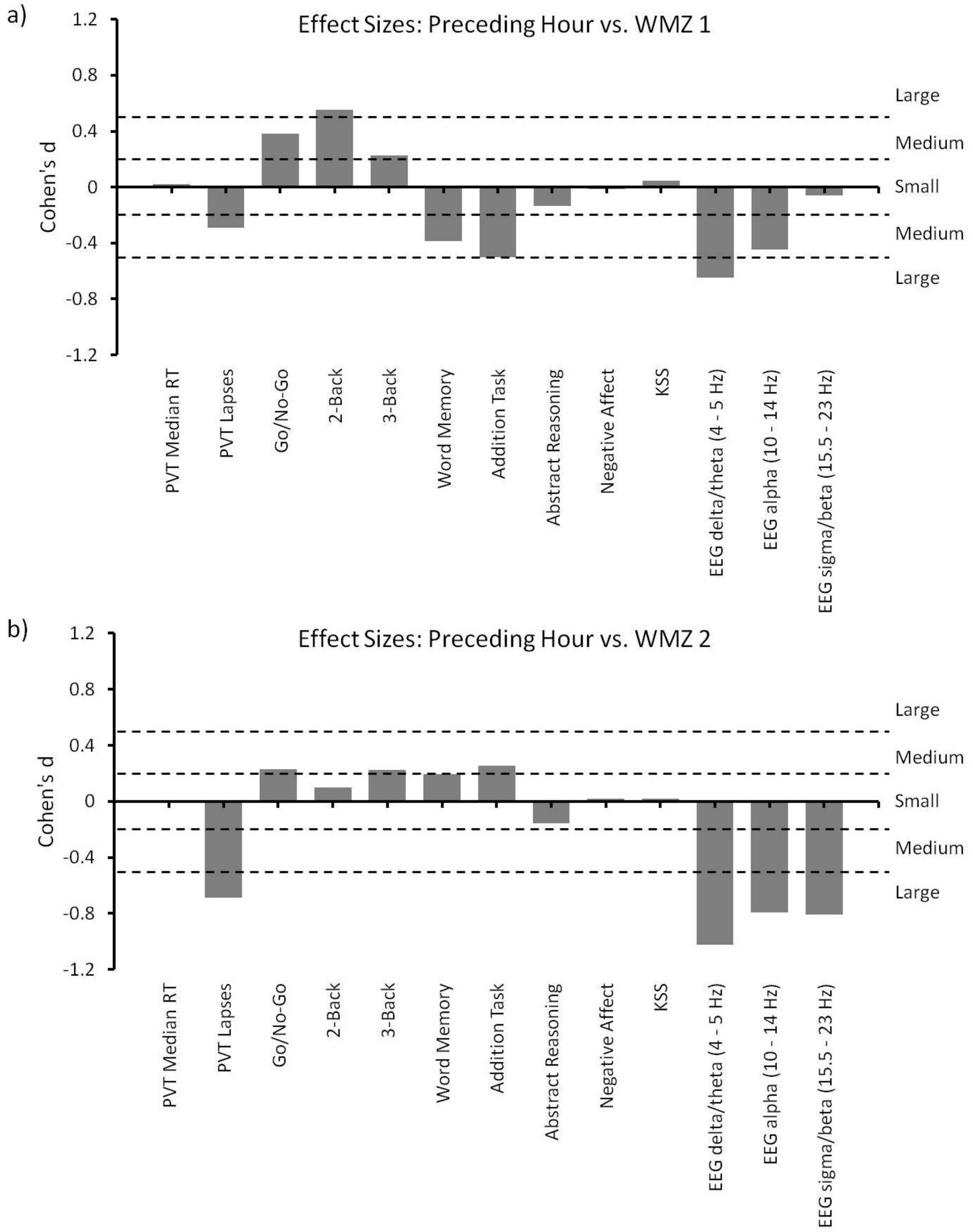

Figure S3. Effect Sizes. Depicted are the effect sizes (Cohen's d) for the comparison of (**a**) WMZ 1 versus its preceding hour and (**b**) WMZ 2 versus its preceding hour. Shown from left to right are: PVT median reaction times in ms; number of PVT lapses; % correct on the Go/No-Go;

% correct on the 2-Back Test; % correct on the 3-Back Test; % correct on the Word-Memory Test; Efficiency on the Addition Task (% correct / reaction time); % correct on the Abstract Reasoning Test; % correct on Negative Affect; subjective sleepiness (KSS); EEG delta/theta activity (4 – 5 Hz); EEG alpha activity (10 – 14 Hz); EEG sigma/beta activity (15.5 – 23 Hz). The dotted lines indicate the cut-offs for the effect size (small < 0.2; medium < 0.5; large > 0.5). A negative effect size indicates a reduction during the WMZ compared to its preceding hour.

Saliva samples

For salivary samples salivettes were used (Salivetten®; Sarstedt Ag & Co.; Nümbrecht; Germany). From these hourly saliva samples (starting 10 min after habitual wake time at the beginning of the CR) the concentration of melatonin was determined with radio-immuno assays by an external laboratory (IBL International GmbH, Hamburg; Germany). The intra-assay coefficients of variability for low- and high-dose control concentration probes were 10.3% and 6.4% (low range: 2.3 – 25.7%; high range: 0.6 – 16.1%). The inter-assay coefficients of variability for low- and high-dose control concentration probes were 12.3 pg/mL and 12.2 pg/mL.
